# Correlating WHO COVID-19 interim guideline 2020.5 and testing capacity, accuracy, and logistical challenges in Africa

**DOI:** 10.11604/pamj.2021.39.89.27522

**Published:** 2021-05-31

**Authors:** Lydia Mosi, Augustina Angelina Sylverken, Kolapo Oyebola, Kingsley Badu, Natisha Dukhi, Nowsheen Goonoo, Priscilla Kolibea Mante, Julien Zahouli, Ebenezer Forkuo Amankwaa, Mai Fathy Tolba, Adeniyi Francis Fagbamigbe, Dziedzom Komi de Souza, Damaris Matoke-Muhia

**Affiliations:** 1 African Academy of Sciences Affiliates, Nairobi, Kenya,; 2 Department of Biochemistry, Cell and Molecular Biology, University of Ghana, Legon, Ghana,; 3 West African Centre for Cell Biology of Infectious Pathogens, University of Ghana, Legon, Ghana,; 4 Kumasi Centre for Collaborative Research in Tropical Medicine, Kumasi, Ghana,; 5 Department of Theoretical and Applied Biology, Kwame Nkrumah University of Science and Technology, Kumasi, Ghana,; 6 Nigerian Institute of Medical Research, Lagos, Nigeria,; 7 Department of Zoology, Faculty of Science, University of Lagos, Lagos, Nigeria,; 8 Human and Social Capabilities Division, Human Sciences Research Council, Cape Town, South Africa,; 9 Biomaterials, Drug Delivery and Nanotechnology Unit, Center for Biomedical and Biomaterials Research (CBBR), University of Mauritius, MSIRI Building, 80837 Reduit, Mauritius,; 10 Department of Pharmacology, Kwame Nkrumah University of Science and Technology, Kumasi, Ghana,; 11 Centre Suisse de Recherches Scientifiques en Côte d´Ivoire, Abidjan, Côte d´Ivoire,; 12 Department of Geography and Resource Development, University of Ghana, Accra, Ghana,; 13 Department of Pharmacology and Toxicology, Faculty of Pharmacy, Ain Shams University, Cairo 11566, Egypt,; 14 School of Life and Medical Sciences, University of Hertfordshire hosted by Global Academic Foundation, New capital city, Egypt,; 15 Department of Epidemiology and Medical Statistics, Faculty of Public Health, College of Medicine, University of Ibadan, Ibadan, Nigeria,; 16 Noguchi Memorial Institute for Medical Research, College of Health Sciences, University of Ghana, Accra, Ghana,; 17 Centre for Biotechnology Research and Development, Kenya Medical Research Institute, Nairobi, Kenya

**Keywords:** COVID-19, SARS-CoV-2, rRT-PCR, testing capacity, diagnostics, Africa

## Abstract

Coronavirus disease 2019 (COVID-19), a severe acute respiratory syndrome caused by SARS-CoV-2 was declared a global pandemic by the World Health Organization (WHO) in March 2020. As of 21^st^ April 2021, the disease had affected more than 143 million people with more than 3 million deaths worldwide. Urgent effective strategies are required to control the scourge of the pandemic. Rapid sample collection and effective testing of appropriate specimens from patients meeting the suspect case definition for COVID-19 is a priority for clinical management and outbreak control. The WHO recommends that suspected cases be screened for SARS-CoV-2 virus with nucleic acid amplification tests such as real-time Reverse Transcription-Polymerase Chain Reaction (rRT-PCR). Other COVID-19 screening techniques such as serological and antigen tests have been developed and are currently being used for testing at ports of entry and for general surveillance of population exposure in some countries. However, there are limited testing options, equipment, and trained personnel in many African countries. Previously, positive patients have been screened more than twice to determine viral clearance prior to discharge after treatment. In a new policy directive, the WHO now recommends direct discharge after treatment of all positive cases without repeated testing. In this review, we discuss COVID-19 testing capacity, various diagnostic methods, test accuracy, as well as logistical challenges in Africa with respect to the WHO early discharge policy.

## Perspective

### Background

The pandemic caused by the Severe Acute Respiratory Syndrome Coronavirus 2 (SARS-CoV-2) continues to spread globally with varying global infection numbers (peaking at over 700,000 per day), leaving over 143 million infected (as of 21^st^ April 2021) with more than 3 million associated deaths [1]. In Africa, as of March 9, 2021, almost four million cases and one hundred thousand deaths had been reported in 55 African union countries [2]. Based on the prevailing local/national policies regarding testing and the general epidemiology of SARS-CoV-2 within different countries, there are different approaches to testing suspected individuals. Suspected cases are usually categorized into 4 groups: (i) symptomatic (individuals with signs or symptoms consistent with COVID-19), (ii) asymptomatic (individuals without visible signs and symptoms of COVID-19 or recent known or suspected exposure to SARS-CoV-2 or those without known or suspected exposure to SARS-CoV-2), (iii) individuals being tested to determine the resolution of infection, (iv) individuals being tested for purposes of public health surveillance for SARS-CoV-2. The viral loads of individuals in each of these categories differ and are directly linked to the amount of exposure and immune status of these individuals [3]. A study has shown that the rate of false negatives is higher when testing is done very early or late during the infection [4]. In many instances, there are significant delays to testing and considering the acute period of the infection, testing samples backlogged beyond three weeks may likely impact accurate tracking of disease timelines and consequent discharge protocol.

Samples used for SARS-CoV-2 diagnosis are collected from the upper or lower respiratory tract and include nasal, nasopharyngeal, oropharyngeal swabs, sputum, lower respiratory tract aspirates, bronchoalveolar lavage, and nasopharyngeal wash/aspirate or nasal aspirate. The viral loads in each of these samples differ both in the stage of the infection and the appropriateness of the sampling technique used [5]. In early infections, upper respiratory tract samples are more suitable for diagnosis while in the late stage of infection, lower respiratory tract specimens are more reliable [6]. Viral loads in stool and urine are comparatively lower [5]. The viral loads within the different samples also differ based on the time taken to process after retrieval with the minimum being 24 hours for biopsies, an average of 2 days for most pharyngeal specimens and up to days for blood, stool and urine. Blood cultures are also useful in testing other complications of the infection. Currently, in Africa, most countries employ “smart screening” approaches with different testing strategies [7] for different purposes: routine surveillance, the port of entry screening and case diagnosis at health centres. There are also different types of tests available or being developed and African countries who are largely plagued with numerous health system challenges have to adapt and localise the specifications of these tests.

In a new policy directive, the WHO recommends direct discharge after treatment of all positive cases without repeated testing. In this review, we discuss local COVID-19 testing capacity in different countries, choice of diagnostic methods, testing accuracy, as well as logistical challenges in Africa with respect to the WHO early discharge policy. We highlight the challenges that are encountered logistically along the global supply chain to get access to testing materials, the differences in the testing methods across different countries and subsequent interpretation of results which ultimately affect testing accuracy. We also point out various challenges faced by African countries to the implementation of all WHO recommendations since the onset of the pandemic, largely due to apparent economic and logistic reasons. These include access to resources for diagnosis, treatment, and control particularly because most of the required logistics are not manufactured by most African countries and have to be shipped or transported from abroad. This article presents a comprehensive discussion on the relevance of the new WHO discharge policy on the inherent and current capacity of public health capacities and Infection Prevention Strategies in different African countries in managing the SARS-CoV-2 pandemic. Finally, the article suggests possible ways in which different countries can mobilize the local and collaborative capacity to adhere to the recommendations by the WHO on public health responses to the pandemic.

### Types of tests for COVID-19

The gold standard in curbing this pandemic is to have as many people tested in order to obtain a fair picture of the rate of infections, identify hotspots and isolate affected individuals in a timely manner for impactful Infection, Prevention and Control measures. In the early months of the COVID-19 pandemic several countries implemented the same lockdown strategies which heavily and negatively impacted on the economy and social lifestyle of many individuals. While this may seem a prudent strategy, there is wisdom in interrogating the impact of such approached to advise future interventions. As is evident, many developed countries are now careful to implement the same level of lockdown in the face of a second wave of infections. Similarly, it was thought early in the pandemic that nose masks were of no use in fighting the spread of the infection. Currently, the wearing of nose masks is strongly enforced. The recommendations by the WHO, whilst carefully crafted, remain guidelines to be implemented as they are or adopted to suit the local needs and policies of countries. Furthermore, the changing nature of policies and recommendations related to COVID-19 demand that any such policy or recommendation be carefully assessed before implementation. There are three main types of COVID-19 tests which include: molecular, antigen and antibody tests as highlighted below and summarized in Table 1.

**Table 1:** summary of diagnostics tests for COVID-19 (adapted from [8])

Test category	Primary clinical use	Specimen type	Performance characteristics	Comments
*NAATs (including RT-PCR)	Diagnosis of current infection	Respiratory tract specimens. (Nasopharyngeal swabs/ washes, oropharyngeal swabs and saliva)	-High analytic sensitivity and specificity in ideal settings. -Clinical performance depends on the type and quality of the specimen and the duration of illness at the time of testing. -Reported false-negative rate ranges from <5 to 40%, depending on the test used.	-Time to perform the test ranges from 15 minutes to 8 hours. -Time to perform the test ranges from 15 minutes to 8 hours. -Turnaround time is influenced by the test used and laboratory workflow. -Some assays allow home collection of specimens that are mailed in.
Serology (antibody detection)	Diagnosis of prior infection (or infection of at least 3 to 4 weeks' duration)	Blood	-Sensitivity and specificity are highly variable. -Detectable antibodies generally take several days to weeks to develop; IgG usually develops by 14 days after onset of symptoms. -Cross-reactivity with other coronaviruses has been reported. -Individual results should be interpreted with caution in settings of low seroprevalence; serologic tests that have high specificity still have a low positive predictive value.	-Time to perform the test ranges from 15 minutes to 2 hours. -Turnaround time is influenced by the test used and laboratory workflow. -It remains uncertain whether a positive antibody test indicates immunity against future infection.
Antigen tests	Diagnosis of current infection	Nasopharyngeal or nasal swabs	-Antigen tests are generally less sensitive than nucleic acid tests. -Sensitivity is highest in symptomatic individuals within 5 to 7 days of symptom onset.	- Time to perform the test is <1 hour.

* NAAT: nucleic acid amplification test; RT-PCR: real-time polymerase chain reaction; IgG: immunoglobulin G

#### (i) Molecular tests

The SARS-CoV-2 genome encodes four structural proteins. The spike surface glycoprotein (S) mediates specific binding to the host cell receptors, the nucleocapsid (N) protein binds to the coronavirus RNA genome to make the nucleocapsid, the membrane (M) protein is the main structural protein that connects between the membrane and the capsid, and the small envelope (E) protein which is involved in the assembly and budding process of the coronavirus [8]. Among them, the genes for the N and E proteins are used as the targets for amplification in the rRT-PCR assay combined with the open reading frame 1 (ORF1) ab, and the RNA-dependent RNA polymerase (RdRP) gene. Nucleic acid amplification tests relying on real-time reverse polymerase chain reaction (rRT-PCR) is the most preferred method for diagnosis and confirmatory of SARS-CoV2 infection [4]. There are several commercially available rRT-PCR kits that supply a variety of premixed solutions for use and each of these has specific protocols [9, 10]. These kits are designed to amplify one and up to three of the four loci of interest in the genome of SARS-CoV2; the nucleocapsid (N), envelope (E), and spike (S) genes, and regions of the RNA-dependent RNA polymerase (RdRp) gene. Depending on the epidemiology of the infection within countries, WHO recommends positive results in at least two loci for highly affected areas and in one locus for areas of low incidence for accurate diagnosis [11]. In addition to rRT-PCR, droplet digital PCR has been tested for COVID-19 diagnosis [4]. Droplet digital PCR has the advantage of absolute quantification and previous studies have shown that it may be more sensitive for virus detection than RT-PCR [12]. Sequencing is highly encouraged to monitor viral genome mutations and molecular evolution; however, this comes with considerable cost implications. Loop-mediated isothermal amplification (LAMP) test is a type of rapid nucleic acid amplification which exhibits increased sensitivity and specificity and does not require expensive reagents or specialized equipment. LAMP is an ultrasensitive nucleic acid amplification method that can be used to detect small numbers of DNA or RNA templates within roughly an hour. The technique is based on the principle of the strand displacement reaction, which occurs under isothermal conditions with the generation of cauliflower-like DNA structures. As the target is recognized by six distinct primers, amplification of a target sequence by the LAMP method is highly specific. It is economical for coronavirus detection [13]. The development of clustered regularly interspaced short palindromic repeats (CRISPR) has led to rapid research expansion and disease detection. To help advance the diagnosis of COVID-19, the CRISPR-based SHERLOCK (Specific High Sensitivity Enzymatic Reporter UnLOCKing) [14] protocol has been developed among others.

#### (ii) Antigen tests

Antigen tests detect the presence of viral proteins in a biological sample, such as saliva or tissue swabbed from the nasal cavity. However, weak signals pose a challenge to antigen tests. Unlike PCR tests which amplify tagged DNA or RNA sequences, making it easy to reliably identify just a few copies of a virus, antigen tests do not amplify their protein signals, so they are inherently less sensitive. Furthermore, the antigen signals get diluted when samples are mixed with liquid needed for capillary action [15].

#### (iii)Antibody (serological) tests

Serological tests are most useful for exposure testing and other epidemiological studies. These immune-based ELISA or lateral-flow assays are useful in measuring levels of IgA, IgG, and/or IgM in serum and have predictive value in diagnosis albeit with low predictive value [16, 17]. The seroconversion rate to produce antibodies following exposure ranges from 1 to 3 weeks and varies significantly amongst individuals [18]. Additionally, cytokine and hemostatic analysis are beneficial for research purposes and understanding of other clinical complications of infection. However, the presence of antibodies does not indicate that an individual is protected from re-infection or has become free of the virus as there is limited understanding of the levels and persistence of antibodies necessary for protective immunity [19]. Antibody tests also are subject to false positives as they can create a positive test result if they react to a different type of coronavirus. Therefore, serology tests may not provide much information about an individual´s infectiousness or otherwise. New rapid point-of-care diagnostic tools now target the virus in saliva or mucus samples from the nose or throat.

### Result interpretation and factors affecting testing accuracy

Real-time Reverse Transcription-Polymerase Chain Reaction methods have an advantage in detecting the SARS-COV-2 infection compared to rapid diagnosis since they detect 2-3 genes. However, there is a possibility of false negatives in the diagnosis given that mutations occur frequently in SARS-CoV-2 which may be a disadvantage of rRT-PCR -based methods. In order to overcome this challenge, it is important to simultaneously use two or more rRT-PCR diagnostic kits that detect different viral genes. Each rRT-PCR kit has a different cycle threshold for determining sample positivity with the ideal ranges between 10 and 45 [20]. Sample positivity is a combination of sample type, the threshold cycle (Ct) value and technicality of sampling technique, stage of infection, the kit type used and number of viral molecular targets (PCR primers). Thus, global standardization is of absolute necessity in differentiating between true positives, false positive and false negatives which range from 5-40% [21].

### Testing capacity and logistical challenges (implementation)

In considering whether the WHO recommendation is the best strategy for Africa, it is important to consider the testing needs and how this is influenced by the logistics, procurement, and testing capacity of different countries. Having considered Africa´s response to COVID-19, it is becoming obvious that the “test, test and test” mantra is not achievable in many African countries as there are significant challenges procuring the necessary reagents and supplies for the needed testing [22]. The dependency of the continent on external suppliers has significantly limited its ability to increase its testing capacity [23]. This is despite the pooled procurement of tests facilitated by the WHO global access to COVID-19 tools [24]. It is important to note that while the majority of cases on the continent are reported in only a few countries, the presumptive low case reportage in other countries is not necessarily due to the absence of cases but the unavailability of the required diagnostic capacity, supplies and the challenges in procurement [25]. As expertly stated by the director of Africa CDC - Dr. John Nkengasong: “If you don´t test, you don´t find”. So far, South Africa and Ghana accounted for nearly half of the testing on the continent [26]. But even in these countries, there are challenges. For example, in Ghana, pooled testing from several cases has been used to conserve testing kits, and it is only when a pool result is positive that samples are followed up individually. Even in Ghana, testing sites acknowledge the limitations associated with the pooling strategy. In South Africa where large-scale community screening and testing were implemented, challenges with massive backlogs and two-week turnaround time for results have been reported, with rippling effects on the health system [22]. Further, Africa´s testing capacity is nowhere compared to other regions. By March 9, 2021, 31.7 million PCR tests for COVID-19 had been conducted in Africa with a population of more than 1 billion people [2]. Europe on the other hand had carried out over 500 million tests [27] for a population of slightly about 741 million as at April 2021

As a result of the pandemic and the need to ramp up testing, Africa has experienced severe logistics challenges in procuring the necessary testing kits for COVID-19, thus lagging in the diagnostic market. “The collapse of global cooperation and a failure of international solidarity have shoved Africa out of the diagnostics market [28]. African countries are therefore struggling to find the tests they need, even when the resources are available [29]. Beyond the logistical challenges linked to getting the tests where needed, other challenges in access to rural and densely populated urban areas, limitations on healthcare personnel and facilities, distrust of healthcare workers, and stigma associated with the virus itself, also affect the ability of African countries to test as many people as possible [30]. While, there are several initiatives through the WHO global pool procurement mechanism [24] or the Africa CDC´s Partnership to Accelerate COVID-19 Testing [23], it is becoming increasingly clear that most countries on the African continent will not be able to circumvent the logistics challenges quickly enough to provide enough testing to all patients (either suspected or recovering from infection). In light of these challenges, we have proposed in Table 2 [31], some corresponding solutions.

**Table 2:** proposed solutions to challenges to COVID-19 testing in African countries

Challenges to COVID-19 testing in Africa	Proposed solutions
Insufficient trained human resource capacity to collect samples and run the tests	Enhance capacity building by leveraging on partner funding and support.
Lack of certified engineers to calibrate and maintain biosafety and laboratory equipment.	Investment in building capacity in local engineers to perform routine equipment maintenance rather than purchasing pricey service contracts.
Lack of health infrastructure, particularly laboratory facilities with bio-safety level class II and higher are very limited.	Expand testing facilities through financial support from international agencies and donors. Encourage the use of alternative and affordable testing methods.
Logistics constraints- almost all consumables for diagnosis, reagents for PCR tests and the test kits are purchased from the international market.	Encourage rapid adoption and validation of point of care tests for diagnosis of COVID-19. African governments should also adopt a fast-track, hassle-free, tax and demurrage exempt plan to facilitate the purchase of reagents and consumables for COVID-19 based research.
Lack of reference laboratories to evaluate testing laboratories.	Establish reference laboratories in African countries which design, implement, and monitor both internal and external quality control tests. This approach is currently being implemented by the WHO for Buruli ulcer diagnosis across endemic countries in Africa [31].
Cultural values- Some people prefer to visit religious and traditional healers rather than modern health care facilities, thereby limiting the number of individuals who undergo COVID-19 testing. Reluctance to undergo testing due to fear of stigmatization. Unwillingness to isolate away from family.	Promote education and communication in local languages using multiple platforms and multiple trusted voices.
Lack of work ethics of health care professionals- Panic, and reluctance to work in SARS-CoV-2 laboratories.	Reassure professionals of their safety through adequate provision of personal protective equipment etc. Encourage mass vaccination of all health care professionals.
Lack of data and weak statistical capacity to accurately account for current cases, circulating strains and predict hotspots for localized lockdowns or quarantines.	Set up of a technology-based data collection system. Strengthen tracking and communication system.

### Resources

Suspected cases of COVID-19 should always be validated by laboratory tests. While RT-PCR still remains the gold standard, issues related to laboratory infrastructure, human resources, supply chain management, and the stockpile of laboratory consumables/reagents need some consideration before Africa adopts the recent WHO discharge protocol or even comes up with a context-specific protocol with the WHO´s as a guideline. Recommendations are that suspected samples are handled under biosafety cabinet, or BSL-2 for RT-PCR) and BSL-3 at the minimum for viral cultures [11]. The infrastructural design of such laboratories not forgetting servicing contracts of equipment may pose a challenge for many African countries since most of these equipments are either assembled or manufactured by and in Western countries. The manufacturers of PCR platforms (such as Roche, Abbott, Hologic, Thermo Fisher, and Cepheid) cannot scale up production quickly enough. And even if they could, chances are that Western countries may be their highest priority. This will mean most cases may not even be sampled let alone tested if these requirements are not met. Beyond infrastructure is trained personnel with the needed expertise and knowledge in not only performing rRT-PCR but importantly, interpreting rRT-PCR results depending on the positivity rates of communities within countries.

The global supply chain is always vital to provide laboratory supplies to ensure timely testing and issuance of test results of suspected cases. However, this is currently heavily fractured and restricted supplies of test reagents and consumables are making it difficult to mobilise capacity. To avert this, efficient humanitarian aid corridors and services to assure the timely provision of laboratory supplies may be an option. A more sustainable option will be a WHO-validated rapid diagnostic test that could provide a faster and cheaper alternative to PCR [11] which can easily be manufactured in African countries considering the continent´s limited manufacturing capacity. While it may be prudent as recommended by the WHO to test suspected cases for other respiratory pathogens using routine laboratory procedures, as recommended in local management guidelines for community-acquired, one wonders how many countries are able to implement this.

### Recommendations in line with an early discharge protocol

The WHO recommendations for early discharge have been proposed after careful consideration of the current global epidemiological state of the SARS-CoV-2 pandemic. They are also largely driven by the economics and other logistical challenges surrounding the Infection Prevention and Control (IPC) strategies within different countries. Africa faces its own sets of challenges compared to other developed nations. The state of the healthcare system, socio-economic and political situation in each African country will inform the level of measures to be implemented. Recent evidence, as this pandemic unfolds, seems to suggest that Africa has been spared the brunt of COVID-19 associated mortalities. In view of this, many countries have started opening gradually to allow some sense of normalcy back. However, all these actions must be done cautiously and in the light of the suggested WHO guidelines. Bearing in mind the science behind the infection during all the stages of infection and associated viral shedding risks, it seems appropriate to maintain the current early discharge regimen.
